# Resilient Membranized Coacervates Formed through Spontaneous Wrapping of Heat‐Destabilized Lipid Bilayers around Coacervate Droplets

**DOI:** 10.1002/advs.202412312

**Published:** 2025-03-21

**Authors:** Sadaf Javed, Evan Spruijt

**Affiliations:** ^1^ Institute for Molecules and Materials Radboud University Heyendaalseweg 135 Nijmegen 6523 AJ The Netherlands

**Keywords:** coacervates, liposomes, protocells, supported bilayers, synthetic cells

## Abstract

Membranes and the membraneless biocondensates help organize cells and work synergistically to drive cellular processes. Separately, membrane‐bound and membraneless compartments face difficulties as stable protocells or synthetic cell systems. Here, we present a new method to create membranized coacervates (MCs) for coacervates with any surface charge and a wide range of phospholipid membrane compositions. MCs are formed when liposomes, destabilized using heat and divalent ions, are mixed with coacervate dispersions. Unlike previous reports of hybrid coacervates surrounded by membranes, the MC membranes form an effective barrier also against small molecules, including calcein and TAMRA. The MC membranes provide excellent stability to the protocells at pH 2–10, salt concentrations of up to 0.5 м, hypotonic and hypertonic conditions, and repeated freeze‐thaw cycles. MCs performed better in all the tested conditions than both coacervates and liposomes. We attribute this behavior to the increased stability that coacervates and liposomes confer to each other when together. MC membranes are unilamellar and fluid, allowing lateral lipid diffusion, and the lipids are more densely packed compared to their corresponding liposomes. MCs can help us understand how stable primitive cells might have emerged and build advanced synthetic cells with enhanced stability and selectivity.

## Graphical abstract

We present a universal way to wrap liposomes around coacervate droplets. Membrane‐enclosed coacervates form when heat‐destabilized liposomes are mixed with coacervates. Membranized coacervates (MCs) can survive pH and ionic strength fluctuations, osmotic stress, and low temperatures. The MC membranes are continuous, unilamellar and fluid, with densely packed lipids. MCs can be used as synthetic cells, cellular delivery vehicles, and protocell models.

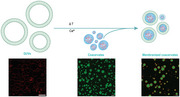

## 1 Introduction

An integral feature of all living matter is the boundary that exists between the internal and the external environments. In modern‐day cells, the cell membrane is an example of such a boundary. Apart from membranes, subcellular compartments, such as biomolecular condensates, help create distinct chemical environments within the cell cytoplasm. Biomolecular condensates are dense, biomolecule‐rich structures formed via liquid‐liquid phase separation (LLPS) of molecules that lack the classic membrane boundary. Nonetheless, the liquid‐liquid interface between biomolecular condensates and the intracellular or intranuclear medium is important for processes, such as, ribosome biogenesis and cellular stress responses.^[^ ^1^ ^]^ Evidently, membranes and biocondensates work synergistically to drive crucial processes, such as mRNA transport,^[^ ^2^ ^]^ signal transduction in T cells,^[^ ^3^ ^]^ autophagy,^[^ ^4^ ^]^ and membrane repair.^[^ ^5^ ^]^

Compartmentalization must have also been a crucial requirement for the persistence and subsequent evolution of early cells, often referred to as protocells. While membrane‐based vesicles and membrane‐free coacervates have both been considered as protocell models, each has its own limitations. Liposomes form a selective barrier between the inside and outside but fail to encapsulate high concentrations of biomolecules in their lumen. They are also prone to bursting or collapsing under osmotic stress. Coacervates, on the other hand, efficiently sequester biomolecules at high concentrations^[^ ^6^ ^]^ but are prone to coalescence and wetting and are sensitive to fluctuations in pH, temperature, and/or salt concentrations. However, different primitive cell‐like entities with diverse structures could have emerged simultaneously through separate processes,^[^ ^7^, ^8^, ^9^, ^10^ ^]^ some closer to condensates while others akin to membranous vesicles. Interactions between clusters of such structures could have led to more complex assemblies, exhibiting a wider range of functions. Ultimately, integrating liposomes and coacervates in one symbiotic system can lead to stable and functional protocells capable of delivering and exchanging molecules across membranes and help us understand how protocells on early earth interacted and evolved.

Studies on biocondensate and membrane interactions in cells have shown that structures comprising the two can be formed without compromising the essential features of the individual components.^[^ ^11^ ^]^ Lu et al. demonstrated that coacervates can wet liposomal membranes to varying degrees and can enter the vesicle lumen via an endocytosis‐like process.^[^ ^12^ ^]^ Both model compartments can remodel each other, often resulting in complex morphologies, such as ruffled or deformed membranes and spreading coacervates.^[^ ^13^ ^]^ While interacting with membranes, coacervates modulate the packing of lipids and their hydration at the coacervate‐membrane junction.^[^ ^14^ ^]^ Coacervate‐membrane interactions are partly based on the ζ‐potentials of coacervates and liposomes and the partitioning coefficients (K_d_) of lipids into the coacervates.^[^ ^15^ ^]^ However, manipulating these interactions to get complex, hierarchical structures remains a challenge.

There have been attempts to create coacervate‐phospholipid vesicle hybrid protocells—either by directly adding lipids solutions, nanoliposomes, or small vesicles to the coacervate solutions.^[^ ^16^, ^17^, ^18^, ^19^, ^20^ ^]^ However, only a little more than half of the coacervates in such cases have continuous membranes with selective permeability that can keep smaller molecules out, and a third of the membranes were permeable to macromolecules (U15).^[^ ^17^ ^]^ As the permeability in such systems depends heavily on the partition coefficient of the client molecule into the coacervate, with a high K_d_ favoring increased membrane permeability,^[^ ^18^ ^]^ the membranes are naturally prone to leakage. Some studies have also induced LLPS within liposomes to form free‐floating coacervates within the vesicles.^[^ ^21^, ^22^ ^]^ In such cases, the formed coacervates do not fill up the entire vesicle lumen and might mimic membraneless organelles in modern cells.

In all the studies mentioned above, the formation of membrane‐enclosed coacervates relied heavily on favorable electrostatic interaction between the coacervates and membranes or lipid solutions for the lipids or liposomes to adsorb on the coacervate surface.^[^ ^16^, ^17^, ^19^ ^]^ Only few coacervate‐membrane combinations form stable hybrid structures; usually because the coacervates are too unstable or lack the appropriate surface charge to interact with the membranes. The range of coacervates that can be used with membranes composed of only zwitterionic lipids is even narrower.^[^ ^20^ ^]^ Among the abovementioned methods, adding lipid solution to coacervates is the easiest way to create a lipid layer around the coacervates. However, free lipids can often partition inside coacervates (Figure S6, Supporting Information) and it is difficult to resolve the final structure and composition of the adsorbed lipid layer.

Here, we present a simple and quick method to create phospholipid membrane‐coated coacervates with a wide range of complex coacervate systems and membrane compositions that is not limited to oppositely charged coacervate‐liposome combinations. When giant unilamellar vesicles (GUVs) are destabilized using a brief heat shock in the presence of divalent ions, they become susceptible to spreading onto surfaces. When they are mixed with coacervates upon cooling, they wrap around the coacervates, forming membranized coacervates (MCs) with intact membranes. We postulate that this wrapping of the liposomal membrane is a bid to stabilize the fissuring liposome using the coacervate as a stabilizing surface, analogous to the spreading of GUV membranes on solid surfaces in the presence of divalent ions.^[^ ^23^ ^]^ We show that the method works for multiple coacervate and membrane compositions, including neutral coacervates and liposomes, and even similarly charged coacervates and liposomes, which otherwise do not interact with each other. The membranes around the MCs are continuous and sufficiently defect‐free to keep small molecules, such as calcein and TAMRA out of the inner coacervate, despite their strong tendency to partition into the bare coacervates. Moreover, the MCs exhibited significantly increased stability compared to both bare coacervates and empty GUVs under changing environmental conditions, such as osmotic shocks and pH fluctuations.

MCs make versatile protocell models with their biomolecule‐rich interior demarcated by a lipid membrane. Our method uses heat and divalent ions to combine phase‐separated droplets with lipid membranes. Such conditions could have easily been present on early earth, making this process a prebiotically plausible way to create protocells. We show that MCs are more stable than coacervates and lipid vesicles, and therefore similar hybrid structures might have been favored on early earth to eventually give rise to modern cells. Due to their enhanced stability, they can be used to study protocell interactions over a wide range of conditions for longer durations compared with the previously reported systems. Moreover, MCs might also lead to a new range of cellular delivery vehicles that sequester high concentrations of substrates and can remain viable over long periods.

## 2 Results and Discussion

### 2.1 A Simple and Robust Method to Create Membranized Coacervates

Inspired by recent studies that demonstrated interactions coacervate‐membrane interactions and membrane remodeling by condensates, we hypothesize that the surface of coacervates could act as template for the assembly of intact lipid bilayer membranes with a wide range of charges and compositions, when provided with a suitable source of membrane fragments and a molecular bridging agent that can mediate the association between coacervate surface and the lipid bilayer. GUVs (1–100 µm in diameter) are a convenient membrane source for the membranized coacervates, as they can be easily visualized under a microscope, allowing us to assess their lamellarity and the presence of lipid aggregates within the vesicle lumen or in solution. Heating GUVs increases the kinetic energy of the constituent lipid molecules and enhances membrane fluidity. Progressive increase in this kinetic energy, through an increase in temperature and duration of heating, will ultimately result in the collapse of the lipid vesicle into an aggregate. However, at temperatures below the tipping point, heating with divalent ions can mildly destabilize the closed bilayer vesicle structure and initiate fissuring of the membrane and the formation of pores without fully collapsing the vesicle.^[^ ^24^, ^25^ ^]^ If the GUV solution is cooled down before the vesicles collapse into aggregates, the vesicles can stabilize themselves, closing the transient pores across the membrane surface.

**Figure 1:**
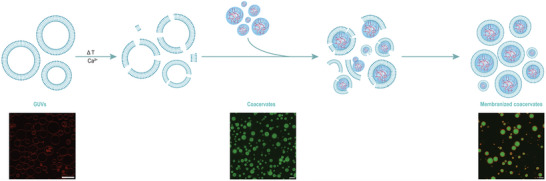
Schematic representation of the process of membranized coacervate formation. In the confocal images, scale bar = 5 µm.

Divalent ions, such as Ca^2+^, adsorb on phospholipid bilayers and interact with the polar head groups of the lipids,^[^ ^26^, ^27^, ^28^, ^29^ ^]^ as well as with phosphate backbone of RNA and polyphosphates that are commonly used to form coacervates. When mildly destabilized vesicles are mixed with coacervates in the presence of divalent ions, they could use the coacervates as a support and wrap themselves around them, analogous to the rupture and fusion of GUVs on solid surfaces to form supported lipid bilayers (SLBs).^[^ ^23^ ^]^ When the coacervate‐adsorbed membrane fragments remain sufficiently mobile, membrane fragments can merge into larger intact membrane sheets by edge fusion, and the membrane may ultimately reseal forming membrane‐enclosed coacervates or membranized coacervates (MCs), analogous to the mechanism underlying SLB formation. **Figure** **1** is a schematic representation of the proposed MC formation process.

**Figure 2:**
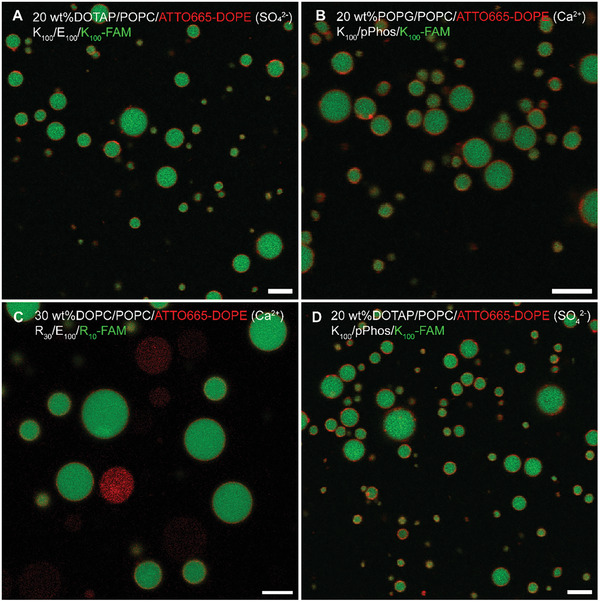
Representative images of membranized coacervates (MCs) formed with different coacervate and GUVs. All GUVs were labelled with ATTO665‐DOPE. Images show MCs formed with A) 20 wt.%DOTAP/POPC GUVs (+) and K_100_/E_100_/K_100_‐FAM coacervates (+) in presence of SO_4_^2−^, B) 20 wt.%POPG/POPC GUVs (−) and K_100_/pPhos/K_100_‐FAM coacervates (−) in presence of Ca^2+^, C) 30 wt.%DOPC/POPC GUVs (neutral) and R_30_/E_100_/R_10_‐FAM (+) coacervates in presence of Ca^2+^, and D) 20 wt.%DOTAP/POPC GUVs (+) and K_100_/pPhos/K_100_‐FAM coacervates (−) in presence of SO_4_^2−^. Scale bar = 5 µm.

We tested this hypothesis by exposing GUVs to a carefully tuned heat shock at 60 °C in the presence of divalent ions (Ca^2+^ or SO_4_^2‐^ at a concentration of 2 mм) of the opposite charge as that of the membrane, or Ca^2+^ in the case of membranes without net charge. We then removed the heat source and mixed the GUVs with a coacervate dispersion, forming membranized coacervates (**Figure** **2**). We verified that addition of 2 mм Ca^2+^ or SO_4_^2‐^ did not dissolve the coacervates (Figure S1, Supporting Information) or destabilize the GUVs (Figure S2, Supporting Information) at room temperature. Furthermore, analysis of the size distributions of the coacervates and GUVs before MC formation, and the formed MCs supports our hypothesis in Figure 1 that the coacervates act as supporting or templating surface for the adsorption and fusion of membrane fragments derived from the GUVs. The size distributions of the MCs and the original coacervates are similar, and significantly different from that of GUVs (Figure S3, Supporting Information). Moreover, addition of divalent ions and heating of GUVs in the absence of coacervates does not lead to a detectable change in GUV size or morphology (Figure S2, Supporting Information).

The procedure we propose does not require monodispersed vesicles, allowing us to directly use the polydispersed GUVs formed using emulsion‐transfer method, bypassing vesicle extrusion. The quality of liposomes affects the quality of the membranized coacervates (MCs): more lipid aggregates in the liposome solution results in non‐uniform membranes around the coacervates (Figure S4, Supporting Information). While the proposed mechanism is not limited to GUVs as membrane source, we found that large unilamellar vesicles (LUVs) are not destabilized in the same way as GUVs upon heat shock with divalent ions. As shown in Figure S5 (Supporting Information), when using 20 wt.% POPG/POPC LUVs to form MCs with the same procedure, we observed large aggregates upon heating at 60 °C with Ca^2+^ and mixing with K_100_/pPhos coacervates. Moreover, due to potential changes in lipid and cholesterol amounts during the extrusion of GUVs to form LUVs,^[^ ^30^ ^]^ we decided to directly use our prepared GUVs to make all subsequent MCs instead of LUVs.

**Figure 3:**
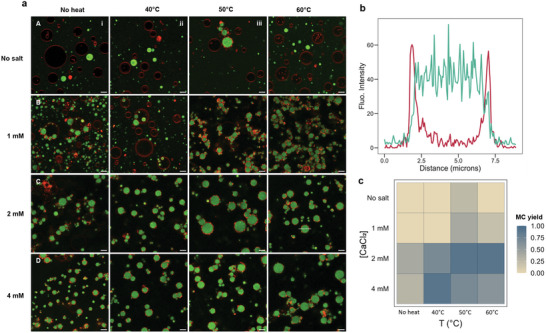
Membranized coacervates form best at temperatures above 50 °C with 2 mM of divalent ions. a) The images show MCs with 20 wt.%POPG/POPC GUVs and K_100_/pPhos/K_100_‐FAM coacervates formed by heating GUVs at 20, 40, 50, or 60 °C, in the presence of 0, 1, 2, or 4 mм Ca^2+^. Without heating the GUVs or in the absence of Ca^2+^, we do not see much interaction between the GUVs and the coacervates. MC formation requires destabilization of GUVs at temperatures above 50 °C in presence of at least 2 mм of divalent ions. b) Representative fluorescence line intensity profiles for an MC along the white dotted line in image C(iv) with coacervate (green) and membrane (red) peaks. c) Heatmap showing the fraction yield of coacervates with distinct, sharp membrane intensity peaks flanking the coacervate intensity peaks measured for n = 10 coacervates in images B(i) through D(iv). For images A(i) through A(iv), line intensities across all coacervates seen in the images were measured. Scale bar = 5 µm.

A key advantage of our method to create MCs is the use of divalent ions as molecular bridging agents between the coacervate surface and the lipid membrane. As divalent ions interact with the polar head groups of many lipids, our method is not limited to combinations of coacervates and membranes of opposite charge, in contrast to previously reported methods to create hybrid coacervate‐vesicle structures (results with lipid solutions in Figure S6, Supporting Information). We confirmed the robustness and versatility of this method to create MCs using multiple coacervate and membrane combinations (Table S1, Supporting Information) and found that it successfully yielded MCs for all the tested combinations, including coacervates and GUVs with very small or no net surface charges (DOPC/POPC), and coacervates and GUVs with similar surface charges (POPG/POPC GUVs and K_100_/pPhos coacervates, and DOTAP/POPC GUVs and R_30_/E_100_ coacervates). This versatility provides flexibility in choosing the best coacervate‐membrane system for downstream application. We imaged the MCs using confocal fluorescence microscopy, shown in Figure 2. It is of particular interest that this method also worked for similarly charged coacervates and GUVs. For example, coacervates formed with poly‐DL‐lysine (K_100_) and polyphosphate (pPhos) have a high negative surface charge (Table S1, Supporting Information).^[^ ^31^ ^]^ If these coacervates are mixed with POPG/POPC GUVs, which also have a net negative charge, the two systems do not interact with each other (as seen in **Figure** **3**A(i)). But heating the POPG/POPC GUVs in the presence of 2 mм Ca^2+^, and then cooling and mixing them with K_100_/pPhos coacervates results in the negatively charged GUVs wrapping around the negatively charged coacervates, forming stable MCs. In such cases, the divalent ions also help screening the surface charge apart from facilitating membrane fissuring.

To further support our hypothesis of the role of temperature and divalent ions in GUV destabilization, we tried making MCs by heating the GUVs at 40, 50, and 60 °C with 1, 2, 4 mм or no divalent ions before mixing them with the coacervates. Ideally, MCs should have a continuous membrane around them and should not agglomerate into clusters. We observed that MCs formed best when GUVs were heated at temperatures above 50 °C with 2 mм of divalent ion salt (**Figure** **3**a,c). Temperatures higher than 60 °C did not yield good MCs (Figure S13, Supporting Information). A higher divalent ion concentration of 4 mM also yielded MCs, even at lower temperatures, however, the formed MCs often agglomerated into large clusters. In addition, lipid particles, which likely originate from small collapsed vesicles, were more abundant on the MC membranes formed at 4 mм divalent ions. Figure 3b shows an ideal fluorescence line intensity profile with the sharp membrane intensity peaks flanking the coacervate intensity signal. We also tried to create MCs in presence of 4, 8, and 16 mм NaCl. If divalent ions were only necessary for charge screening (in case of similarly charged coacervates and GUVs), then monovalent ions at equivalent ionic strength should also facilitate MC formation. However, we did not observe MCs after heating the GUVs with NaCl and mixing them with the coacervates (Figure S7, Supporting Information). Finally, we tested other divalent ions (Mg^2+^ and Fe^2+^) to form MCs (Figures S14 and S15, Supporting Information). While we did see some membranes around the coacervates, they were not continuous, presumably because the interactions between Mg2+ and Fe2+ and the coacervates was too strong (Figure S14, Supporting Information). We think that the salt concentration and heating temperatures can be adjusted to optimize them for other divalent ions.

### 2.2 MC Membranes Around Coacervates Have Similar Barrier Function as Phospholipid Bilayers in GUVs

**Figure 4:**
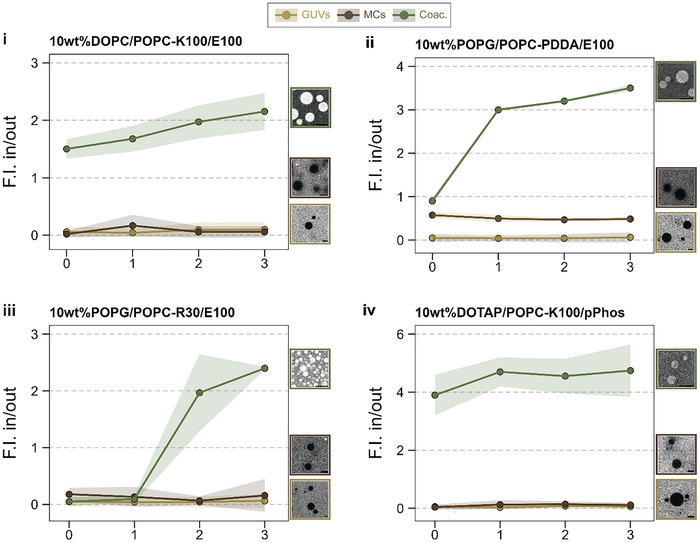
Membranized coacervate membranes form a selective barrier, keeping small molecules out similar to GUV membranes. The plots show ratios of fluorescence intensities inside giant unilamellar vesicles (GUVs), membranized coacervates (MCs), and coacervates (Coac.) over that in solution for calcein (i, ii, and iii) and 5‐TAMRA (iv) over time. Each point represents the mean intensity ratios for n = 5 circular ROIs inside and outside each species. The shaded ribbon along the points indicates the standard deviation. Scale bar in the images = 5 µm.

Confocal microscopy images of MCs indicated that there is a continuous membrane around the coacervates. However, we wanted proof that the membrane forms a robust barrier between the inner coacervate phase and the outer dilute phase. To test the permeability of the membrane, we monitored the diffusion of calcein and 5‐TAMRA (both used at a final concentration of 2 mм) across the MC membrane and compared it with their diffusion across GUVs and bare coacervates of the same composition. Calcein does not have partition inside K_100_/pPhos, likely due to the repulsion between the negatively charged pPhos and calcein molecules. Therefore, we used 5‐TAMRA for K_100_/pPhos coacervates and the corresponding MCs and GUVs to compare the partitioning of the dye between MCs, GUVs, and coacervates (**Figure** **4**(iv)). Coacervates, due to their lesser water content (or reduced polarity) compared to the surrounding dilute aqueous phase, have high affinity for hydrophobic molecules.^[^ ^32^ ^]^ This means, that if the membrane around the coacervate is not continuous, most fluorescent dyes will rapidly partition inside the coacervate.

As expected, the dyes swiftly partitioned inside the coacervates (Coac.), resulting in higher fluorescence intensity inside the coacervate compared to the outside. By contrast, the MC membranes kept the small molecules out and the fluorescence intensity in the coacervate interior remained low, similar to that in the GUV lumen. Figure 4 shows the increase in the ratio of fluorescence intensities inside the GUVs, coacervates, and MCs over outside with time. The intensity ratio for MCs remained nearly constant for more than 3 hours, indicating that the MCs did not take up the dyes over time. In case of MCs formed with 10 wt.% POPG/POPC GUVs and PDDA/ATP coacervates (Figure 4(ii)), the fluorescence intensity ratio is slightly larger than that for the corresponding GUVs but remains constant over time. This could be because the membrane fragments around the coacervate took longer to close the gaps between each other and some calcein diffused inside the coacervates before all the defects could seal. Once the membrane fragments merged into a continuous unit, the diffusion of calcein stopped and the fluorescence intensity ratio in/out stayed constant over time. Hence, the membranes around the MCs are continuous and form a selective barrier.

As a control, we added melittin (final concentration 10 µg mL^−1^)—the pore‐forming bee venom peptide—to the POPG/POPC‐K_100_/pPhos MC solution containing 5‐TAMRA to check whether melittin can permeabilize the membrane. The MC membranes became permeable to 5‐TAMRA after melittin addition (Figure S8, Supporting Information), indicating that MC membranes are comparable in thickness to single lipid bilayers, and they are not multilamellar in nature.

### 2.3 MCs are Resilient to Fluctuations in the External Environment

**Figure 5:**
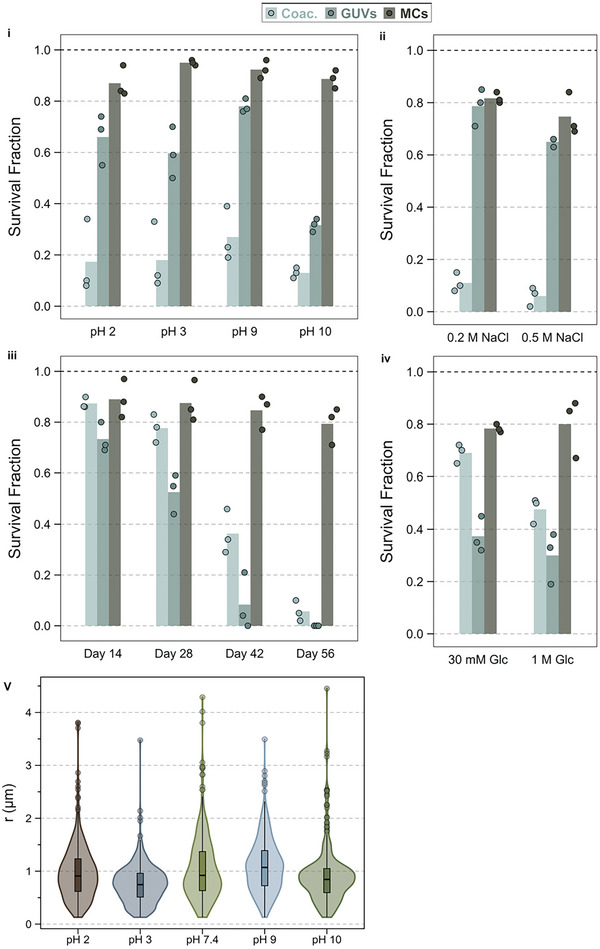
MCs are more resilient to environmental fluctuations compared to GUVs and coacervates. The plots show the survival fractions of K100/pPhos‐20 wt.%POPG/POPC membranized coacervates (MCs), giant unilamellar vesicles (GUVs), and coacervates (Coac.) under changing pH (i), increasing salt concentrations (ii), through freeze‐thaw cycles (iii), and osmotic stress (iv) compared to the MCs in the optimal solution conditions (pH 7.4, no salt, 300 mм glucose; survival fraction = 1). MC size distribution at different pH (v).

Protocells must have needed to remain stable or viable under a range of environmentally changing conditions to survive and evolve. Vesicles and coacervates have different strengths and weaknesses—the former are prone to bursting or collapsing under osmotic stress, while the latter are sensitive to high salt concentrations or extreme pH. We hypothesize that MCs combine the strengths of both protocell systems and remain stable under a much wider range of conditions. To test this hypothesis, we subjected the MCs various environmental stresses—changes in pH, osmolarity, and salt concentrations (**Figure** **5**; Figure S10, Supporting Information). We also tested their viability over time by freezing the MC dispersions at −20 °C and thawing them repeatedly.

First, we tested the resilience of MCs to changes in pH. Most complex coacervates contain components that can be protonated or deprotonated in acidic or basic conditions. When formed in buffer‐less solutions at physiological pH (7.3–7.4), these coacervates cannot withstand large pH fluctuations. pH fluctuations can also deform or induce chemical polarization in lipid vesicles and lead to bursting.^[^ ^33^ ^]^ We exposed MCs made from K_100_/pPhos coacervates and 20 wt.% POPG/POPC GUVs to pH 2, 3, 9, and 10 by adding 0.1 м HCl or 0.1 м NaOH to the solution and monitored their structural integrity over time in comparison to the same MCs at pH 7.4 (control) by counting the number of MCs, GUVs, or coacervates and measuring their radii in the control versus the stressed samples. Figure 5(i) shows that MCs weathered the stresses due to pH variations well. At pH 2, 3, 9, and 10 the fractions of MCs surviving compared to the control (survival fraction = 1) were 0.86 ± 0.05, 0.95 ± 0.01, 0.92 ± 0.02, and 0.89 ± 0.03, respectively. GUV fractions at pH 2, 3, 9, and 10 compared to that at pH 7.4 were 0.65 ± 0.08, 0.59 ± 0.08, 0.78 ± 0.02, and 0.31 ± 0.02, respectively. Meanwhile, the fractions of coacervates surviving at pH 2, 3, 9, and 10 were 0.14 ± 0.12, 0.15 ± 0.1, 0.26 ± 0.09, and 0.13 ± 0.02, respectively. The MC sizes were also comparable in different conditions (Figure 5(v); Figure S9, Supporting Information).

We also tested the effect of 200 and 500 mм NaCl on MC stability. MCs were able to withstand changing salt concentrations with survival fractions at 200 and 500 mм NaCl of 0.82 ± 0.02 and 0.76 ± 0.06, respectively (Figure 5(ii)). GUVs also fared comparably well, with survival fractions of 0.74 ± 0.04 and 0.6 ± 0.06 at 200 and 500 mм NaCl, respectively. Coacervates, on the other hand, dissolved and rapidly coalesced at the tested salt concentrations with 0.14 ± 0.06 and 0.06 ± 0.04 of them surviving compared to those in salt‐less solutions. We attribute the enhanced resilience of MCs to changes in salt concentration to the continuous membrane surrounding the coacervates. If the MC membrane was not continuous, the coacervate polymers would leak out of the MCs once the salt ions disrupted the electrostatic interaction between the oppositely‐charged polymers, resulting in dissolution of the MC core.

To test their resilience to freeze‐thaw cycles, we stored MCs in an eppendorf at ‐20 °C, thawed them every two weeks for eight weeks, and assessed their number and morphology. The fractions of MCs surviving after two, four, six, and eight weeks were 0.89 ± 0.06, 0.87 ± 0.07, 0.84 ± 0.05, and 0.79 ± 0.06, respectively (Figure 5(iii)). We did not observe visible changes in the MC morphology. By contrast, less than 10% of bare coacervates and liposomes were still intact after eight weeks of freeze‐thaw cycles.

Finally, we exposed MCs, coacervates and liposomes to hyper and hypo‐osmotic shocks. Liposomes are sensitive to differences in osmolarities between the inner and outer solutions. In hypotonic conditions, the vesicles swell and eventually burst. Under hypertonic stress, water diffuses out of the vesicles making them flaccid and unstable. We subjected MCs made in 300 mм glucose solution to hyper and hypo‐osmotic stress by suspending them in 1 м and 30 mм glucose solutions, respectively, and monitored their stability over time. The MC survival fractions in 30 mм and 1 м glucose were 0.78 ± 0.01 and 0.79 ± 0.1, respectively. Meanwhile, liposomes of the same composition either burst in hypotonic solution (survival fraction = 0.35 ± 0.07) or collapsed in hypertonic solution (survival fraction = 0.31 ± 0.09), resulting in lipid aggregate deposition at the substrate (Figure 5(iv)). Coacervates fared better in osmotic stress than the liposomes with survival fractions of 0.69 ± 0.03 and 0.49 ± 0.05 in hypo‐ and hyperosmotic stress, respectively. Our results indicate that MCs are much more stable than their constituent species and can withstand drastic changes in the external environment. These features make MCs an ideal protocell model and synthetic cell compartment to encapsulate and store molecules for extended periods, carry out reactions, and host complex reaction networks in a well closed‐off and distinct environment.

### 2.4 Lipids in MC Membranes are Mobile but Densely Packed

**Figure 6:**
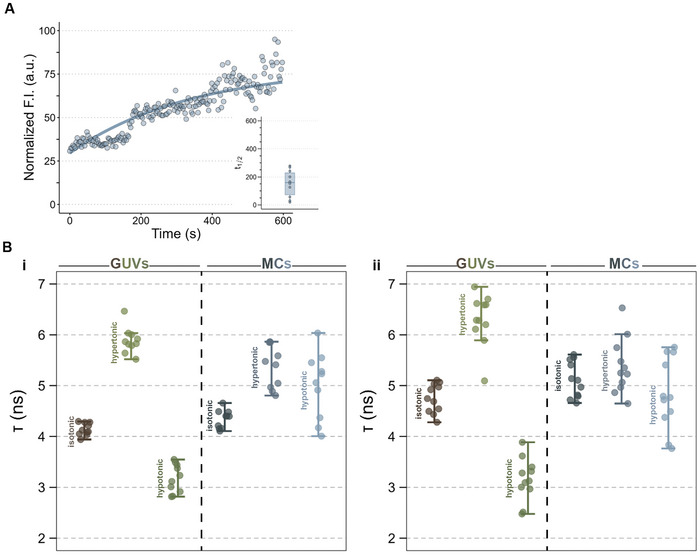
Properties of the MC membranes. A) A representative fluorescence recovery curve of an ROI in K100/pPhos‐20 wt.%POPG/POPC MC membrane labelled with ATTO633‐DOPE; the inset shows the half times of fluorescence recovery for ROI in n = 10 MC membranes. B) Flipper‐TR™ fluorescence lifetimes in 20 wt.%POPG/POPC GUVs and K100/pPhos‐20 wt.%POPG/POPC MC membranes containing 10 wt.% (i) or 20 wt.% (ii) cholesterol in isotonic (300 mM glucose), hypertonic (500 mM glucose), and hypotonic (200 mM glucose) conditions.

We also monitored the fluorescence recovery after photobleaching (FRAP) to determine the mobility of the lipids in K100/pPhos‐20 wt.%POPG/POPC MC membranes. The ease of lipid diffusion across the membrane also gives an idea about the membrane continuity. If the lipid bilayer is discontinuous or the lipids are adsorbed on the surface of the coacervates without forming a well‐defined bilayer, then lateral diffusion of the lipids will be slow or nearly absent and we will see little to no fluorescence recovery. **Figure** **6**A shows a representative FRAP curve and the half‐time of recovery (t_1/2_) we obtained for our MCs (n = 10). Most MC membranes showed a fluorescence recovery of ≈70% of the pre‐bleach levels, indicating that the lipids in the MC membranes remain mobile. We noticed that the t_1/2_ varied widely across different MCs. We think that this might be because the MC membrane and the coacervate interact with each other rather than co‐existing as separate entities in the MC structure. In that case, the material properties of the coacervate and the strength of positive interaction between the MC membrane and the coacervate will influence the lipid mobility in the MC membrane, resulting in wide variations in lipid mobility between MCs or even between different regions of one MC membrane.

Finally, we also assessed the lipid packing within the MC membranes by monitoring the changes in the fluorescence lifetime (τ) of an environment‐sensitive probe, Flipper‐TR,^[^ ^34^ ^]^ using fluorescence lifetime imaging microscopy (FLIM) (Figure 6, S12). In membranes with high surface tension, loose lipid packing, or low cholesterol content (fluid membranes), Flipper‐TR has shorter τ as the rotor molecule largely exists in a non‐planar form.^[^ ^34^ ^]^ However, in membranes with low surface tension, denser lipid packing, or high cholesterol content (rigid membranes), the molecule is sterically hindered and exists in its planar form with longer τ.

We subjected GUVs and MCs with the same membrane composition to hyper and hypoosmotic conditions and measured the fluctuations in Flipper‐TR τ (Figure 6B). We used 20 wt.%POPG/POPC GUVs with 10 wt.% (Figure 6B(i)) and 20 wt.% (Figure 6B(ii)) cholesterol to make MCs with K100/pPhos coacervates. As expected, Flipper‐TR in membranes with 20 wt.% cholesterol had longer τ values (4.74 ± 0.29 and 5.1 ± 0.35 ns for GUVs and MCs, respectively, in 300 mм glucose solution) compared with that in membranes with 10 wt.% cholesterol (4.14 ± 0.12 and 4.16 ± 0.25 for GUVs and MCs, respectively, in 300 mм glucose solution).

MC membranes have a denser lipid packing compared to their GUVs counterparts as indicated by the longer τ values for MCs in isotonic conditions compared with that of GUVs of the same composition. In the case of GUVs, the τ values varied depending on the solution osmolarity, indicating that the surface tension and the lipid packing of the GUV membrane fluctuates and is largely dependent on the external environment. In case of MCs, we see more variability in the τ values, even within the same solution conditions. This further supports the hypothesis that the membrane properties are influenced by the coacervate interiors, resulting in variability in lipid packing from MC to MC. However, the τ values for MCs in isotonic (300 mм glucose), hypertonic (500 mм glucose), or hypotonic conditions (200 mм glucose) do not show discernible differences as they do for the corresponding GUVs (Table S2, Supporting Information). This indicates that MC membranes do not undergo large changes in their lipid packing in response to osmotic changes, likely due to the support of and interaction with the coacervate interior. This symbiotic relationship between the membrane and the coacervate also explains the increased stability of MCs under stress conditions.

## 3 Conclusion

While coacervates and liposomes can mimic certain components of modern cells—biocondensates and membrane‐bound compartments, respectively—and hence have served as protocells models, neither can epitomize living cells alone. Here, we have shown a simple and robust method to create membranized coacervates with a biomolecule‐rich interior delineated by a lipid membrane. MCs are more resilient against changing environmental pH, osmolarity, and salt concentrations and can withstand multiple freeze‐thaw cycles, making their long‐term storage feasible. The MC membranes are continuous, their lipids more densely packed than the corresponding GUVs, and their fluidity is influenced by the inner coacervate core. This preparation method for MC is simple and prebiotically plausible using only heat and divalent ions to combine phospholipids bilayers and coacervates in a single structure. We can use these MCs as protocell models and study interactions and biomolecule exchange between various MC populations to better understand how primitive cells might have interacted and evolved. Moreover, MCs might also serve as stable and versatile cellular delivery vehicles.

## 4 Experimental Section

### Lipids

All unlabelled lipids: 1‐palmitoyl‐2‐oleoyl‐sn‐glycero‐3‐phospho‐(1′‐racglycerol) (POPG), 1,2‐dioleoyl‐sn‐glycero‐3‐phosphocholine (DOPC), 1,2‐dioleoyl‐3‐trimethylammonium‐propane (DOTAP), and 1,2‐dioleoyl‐sn‐glycero‐3‐phosphocholine (DOPC) were purchased from Avanti Polar Lipids dissolved in chloroform (25 mg mL^−1^). The chloroform was evaporated, and the lipids were redissolved in half of the initial volume of chloroform to obtain 50 mg mL^−1^ lipid stock solutions. The stock solutions were stored at −20 °C until used. 1,2‐Dioleoyl‐*sn*‐glycero‐3‐phosphoethanolamine (DOPE) conjugated to ATTO665 or ATTO633 was purchased from ATTO‐TEC GmbH in powder form and used to label the GUVs. The powder was dissolved in an appropriate volume of chloroform to form 1 mg mL^−1^ stocks of the labelled lipids.

### Coacervate Components

K_100_, E_100_, R_30_, and R_10_ were purchased from Alamanda Polymers and dissolved in MQ at stock concentrations of 50 mg mL^−1^, 50 mg mL^−1^, 0.10 м [monomer], and 0.10 м [monomer], respectively. Hexametaphosphate, poly(diallyldimethylammonium chloride) (PDDA), ATP, and polyuridylic acid (potassium salt) were purchased from Sigma–Aldrich and dissolved in MQ at stock concentrations of 50 mg mL^−1^, 50 mg mL^−1^, 50 mM, and 10 mg mL^−1^, respectively. (RGRGG)_5_ and R_10_‐FAM were purchased from Genscript and dissolved in MQ at stock concentration of 0.10 м [monomer].

### Coacervate Formation

Coacervates were made by depositing the polycation and polyanion solutions on the wall of the Eppendorf tube, containing Milli‐Q, close to the meniscus. The final volume of the solution was 20 µL. The tube was then vortexed, introducing the polymers in water to form coacervates in solution with the desired final concentrations. The charge concentrations of the final coacervate dispersions was adjusted by changing the mixing ratios of the polycation and polyanion as required.

### GUV Formation

GUVs were created using the emulsion‐transfer method.^[^ ^35^ ^]^ Briefly, lipid stock solutions (12 µL, final lipid concentration 1.5 mg mL^−1^) were added in paraffin oil. Cholesterol was added at a final concentration of 10 wt.% or 20 wt.% (relative to the lipids), and DOPE‐PE and ATTO‐DOPE labels were added at a final concentration of 0.17 wt.% (relative to the lipids). The final volume of the lipid mixture was 400 µL. The mixture was heated at 80 °C on a thermoshaker for 5 min to evaporate the chloroform under constant nitrogen gas flow to reduce the effects of humidity on the liposome formation. Then, the oil‐lipid mixture was sonicated for 15 min in a bath sonicator. To make the emulsion, sucrose was added to oil‐lipid mixture to a final concentration of 300 mм and the tubes were agitated. After incubating the emulsion for 10 min, it was layered over glucose (400 µL, 300 mм) in another tube. These tubes were then centrifuged at 9000 rcf at 4 °C for 30 min. After centrifugation, the bottom of the tubes were punctured using a needle and the pellets deposited in the aqueous phase were collected into new tubes. The transferred suspension was centrifuged again at 6000 rcf at 4 °C for 10 min. The supernatant was removed from the tubes and the pellet was resuspended in fresh 300 mм glucose. The GUVs were then visualized under a confocal microscope.

### MC Formation

To make the MCs, we first added the appropriate divalent salt (CaCl_2_ or Na_2_SO_4_, at a final concentration of 2 mм) to the GUV solution in a tube. Then, we made the coacervate solution by mixing the polycation and polyanion in glucose (300 mм). The GUV solution was placed on a thermoshaker and heated at 60 °C for about a minute. The GUV solution was cooled down for 5–10 s before the coacervate solution was added. Alternatively,  the GUV solution can be placed on ice with immediate addition of the coacervate solution. The GUV‐coacervate mixture was then vortexed thoroughly for 30 s. The obtained MCs can be then visualized under a microscope and used as desired. Note: The coacervate solution must be added within a few seconds of taking the GUVs out of the thermoshaker as GUVs can stabilize back into intact vesicles upon complete cooling.

### Membrane Permeability Assays

After preparing the MCs, we added 20 µL each of the GUV, coacervate, and MC solutions in separate wells of 18‐flat well µSlide (Ibidi). Then, calcein (2 mм) was added in each well. To compare dye partitioning between GUVs, MCs, and bare K_100_/pPhos coacervates coacervates, we used 5‐TAMRA (2 mм) as calcein does not partition in K_100_/pPhos coacervates. The samples were allowed to rest for 10 min before imaging. For all samples, we measured the average fluorescence intensities of five circular regions of interest (ROIs, area = 1.34 µm^2^) inside the GUVs, coacervates, or MCs and in the solution to obtain the fluorescence intensity ratios.

To permeabilize the MC membranes, we added melittin (10 µg mL^−1^) (Sigma–Aldrich Chemie, Merck Life sciences N.V.) to MC suspension containing the fluorescent dyes. The samples were in cubatedfor 10 min before imaging.

### MC Resilience Experiments

For each replicate, the MC, GUV, and coacervate solutions were split into parts (20 µL each). To test the MCs, GUVs, and coacervates in different pH, the pH of the dispersions were adjusted to 2, 3, 9, or 10 using HCl (0.1 м) or NaOH (0.1 м). To test the MCs, GUVs, coacervates at different salt concentrations, NaCl (200 or 500 mм) was added to the samples. To expose the MCs, GUVs, and coacervates to osmotic stress, glucose (final concentration 1 м) or MilliQ (to dilute the final glucose concentration to 30 mм) was added to the solutions. To test the viability of MCs, GUVs, and coacervates through multiple freeze‐thaw cycles, the aliquots of each species were stored at −20 °C for two, four, or eight weeks.

We captured ten confocal images each for MC, GUV, and coacervate samples per replicate. Then, we counted the number of particles in each image to get the total number of particles across the ten captured images per sample either manually (for GUVs) or using the EBImage package (for MCs and coacervates) in R (Bioconductor).^[^ ^36^ ^]^ This was performed in three independent experiments for all the MC, GUV, and coacervate samples in different conditions. The controls for osmotic stress, salt concentrations, pH, and freeze‐thaw cycles were freshly prepared MCs, GUVs, or coacervates present in glucose (300 mм) without NaCl at pH 7.4. The survival fraction of MCs, GUVs, or coacervates was calculated as follows:

(1) $$Survival\;Fraction=\frac{\sum_{i=1}^{10}\left(treatment\right)}{\sum_{i=1}^{10}\left(control\right)}$$

The size distribution of MCs, GUVs, and coacervates in different condition was also measured either manually (for GUVs) or using the EBImage package (for MCs and coacervates) in R.

### Image Acquisition

All confocal images were captured using a Leica SP8x confocal inverted microscope equipped with a DMi8 CS motorized stage, a pulsed white light laser, two HyD SP GaAsP and two PMT detectors. Images were recorded using a 63x HC PL APO oil immersion objective.

FRAP experiments were performed using a Leica SP8 SMD microscope with a HCX PL APO CS 63.0×1.20 WATER UV objective. The membrane was labelled with ATTO633‐DOPE. A constant ROI area of 1.34 µm^2^ for all measurements was used. The same ROI area was used to measure the control (a different region on the same MC membrane) and background regions. The duration between each image acquisition was 3 s for all measurements and 20 frames were recorded before bleaching the ROI. The ROI was bleached with 10 pulses of 544 nm argon laser at 100% intensity and 50% power, and the recovery was monitored using 633 nm excitation laser at 0.5% intensity (50% power) and the fluorescence detected using a PMT detector in the range of 645–670 nm. The fluorescence intensities were normalized using the following formula:^[^ ^37^ ^]^

(2) $$F{\left(t\right)}_{norm}=100\times \frac{F{\left(t\right)}_{ROI}-F{\left(t\right)}_{bkgd}}{F{\left(t\right)}_{ctrl}-F{\left(t\right)}_{bkgd}}\times \frac{F{\left(t\right)}_{pre-ctrl}-F{\left(t\right)}_{bkgd}}{F{\left(t\right)}_{pre-ROI}-F{\left(t\right)}_{bkgd}}$$

where, *F*(*t*)_*ROI*_ is the fluorescence intensity of the ROI, *F*(*t*)_*ctrl*_ is the fluorescence intensity of the control region, *F*(*t*)_*bkgd*_ is the fluorescence intensity in solution at time t, and *F*(*t*)_*pre* − *ctrl*_ and *F*(*t*)_*pre* − *ROI*_ are the pre‐bleach intensities of the control region and the ROI, respectively. The fluorescence intensity recovery curve was fitted using FRAPbot (v. 1.9)^[^ ^38^ ^]^ to obtain the t_1/2_ values.

FLIM imaging was performed using Leica SP8 SMD microscope equipped with internal Leica HyD (Hybrid) detectors and Falcon FLIM software. The sample was excited using 488 nm pulsed argon laser operating at 10 MHz, and the emission signal was collected using HyD detector from 550–650 nm in photon counting mode. The instrument response function (IRF) was measured to be 142.9 ps (measured using 1 µM fluorescein quenched using 3 м KI in 300 mм glucose solution). The fluorescence lifetime decay curves (for individual MCs and GUVs) were fitted to a double‐exponential model using FLIMfit.^[^ ^38^ ^]^ The initial tau values were set to 2 ns. Out of the two lifetime components, τ_1_ was the longer component with higher photon counts, and was used to represent the lifetime data.

For hypotonic stress, the GUVs and the corresponding MCs solutions were diluted from an initial glucose concentration of 300 mм to a final concentration of 200 mM. For hypertonic stress, 3 м glucose solution was added to GUVs and MCs solutions to get a final concentration ≈500 mM. Flipper‐TR probe (Spirochrome) was added to each sample at a final concentration of 2 µm.

### Image Analysis

Images were processed using ImageJ (FIJI). The brightness and contrast were auto‐adjusted to aid visualization in the figures. The fluorescence intensity profiles were plotted in R. For Figure 3 panel A, the line intensities in the coacervate and membrane channels for all the visible coacervates were measured. For panels B, C, and D, the line intensities for n = 10 species in the coacervate and membrane channels were measured. The number of species with two sharp membrane signal peaks flanking the coacervate signal peak were counted and their fraction among the total number of species assessed (n = 10) was obtained. The coacervates showing sharp membrane peaks if they were attached to liposomes were excluded from this fraction. We also excluded the coacervates from the MC count if they had significant membrane signal inside the coacervates in addition to the two flanking membrane peaks.

## Conflict of Interest

The authors declare no conflict of interest.

## Supporting information



Supporting Information

## Data Availability

The data that support the findings of this study are available in the supplementary material of this article.
