# Supplementary material for: Resilient Membranized Coacervates Formed through Spontaneous Wrapping of Heat‐Destabilized Lipid Bilayers around Coacervate Droplets
Source: Adv Sci (Weinh). 2025 Mar 21;12(19):2412312. doi: 10.1002/advs.202412312 (PMC12097097; doi:10.1002/advs.202412312)
Supplement: Supplementary file 1 — Supporting Information [file ADVS-12-2412312-s001.pdf]

ADVANCED SCIENCE
Open Access

Supporting Information

for *Adv. Sci.*, DOI 10.1002/advs.202412312

Resilient Membranized Coacervates Formed through Spontaneous Wrapping of Heat-Destabilized Lipid Bilayers around Coacervate Droplets

*Sadaf Javed and Evan Spruijt**

Supporting information

Resilient Membranized Coacervates Formed Through Spontaneous Wrapping of Heat-Destabilized Lipid Bilayers Around Coacervate Droplets

Sadaf Javed [a], Evan Spruijt*[a]

[a] *Institute for Molecules and Materials, Radboud University, Heyendaalseweg 135, 6523 AJ Nijmegen, The Netherlands.* * E-mail: e.spruijt@science.ru.nl

Table S1: GUVs and coacervates used to create membranized coacervates, their mixing charge concentration ratios, and ζ-potentials (mV).

GUVs		Coacervates			
Composition	ζ-potential (mV)	Polyanion	Polycation	Mixing [charge] ratio (coacervates)	ζ-potential (mV)
30wt% DOPC/POPC	-9.6	Poly-DL-Glutamate (x=100)	Polyarginine (x=30)	2:1	15.8
30wt% POPG/POPC	-27.1	Hexametaphosphate	Poly-DL-Lysine (x=100)	2:1<br>1:1	-26.4<br>-12.6
10wt% DOPC/POPC	n.a.	Poly-DL-Glutamate (x=100)	Poly-DL-Lysine (x=100)	1:1	17.0
10wt% DOTAP/POPC	3.4	Poly-DL-Glutamate (x=100)	PDDA	1:3	35.7
10wt% POPG/POPC	-8.8	Poly(uridylic acid) (x=15)	Poly-DL-Lysine (x=100)	0.65:1	42.6
30wt% DOPC/POPC	-9.6	ATP	PDDA	2:1	22.1
10wt% DOTAP/POPC	3.4	Hexametaphosphate	Poly-DL-Lysine (x=100)	2:1	-26.4
30wt% DOTAP/POPC	18.9	Poly-DL-Glutamate (x=100)	Polyarginine (x=30)	2:1	15.8
20wt% POPG/POPC	-15.8	Poly-DL-Glutamate (x=100)	Polyarginine (x=30)	2:1	15.8
20wt% DOTAP/POPC	6.1	Poly-DL-Glutamate (x=100)	$(RGRGG)_5$	1:2	12.5

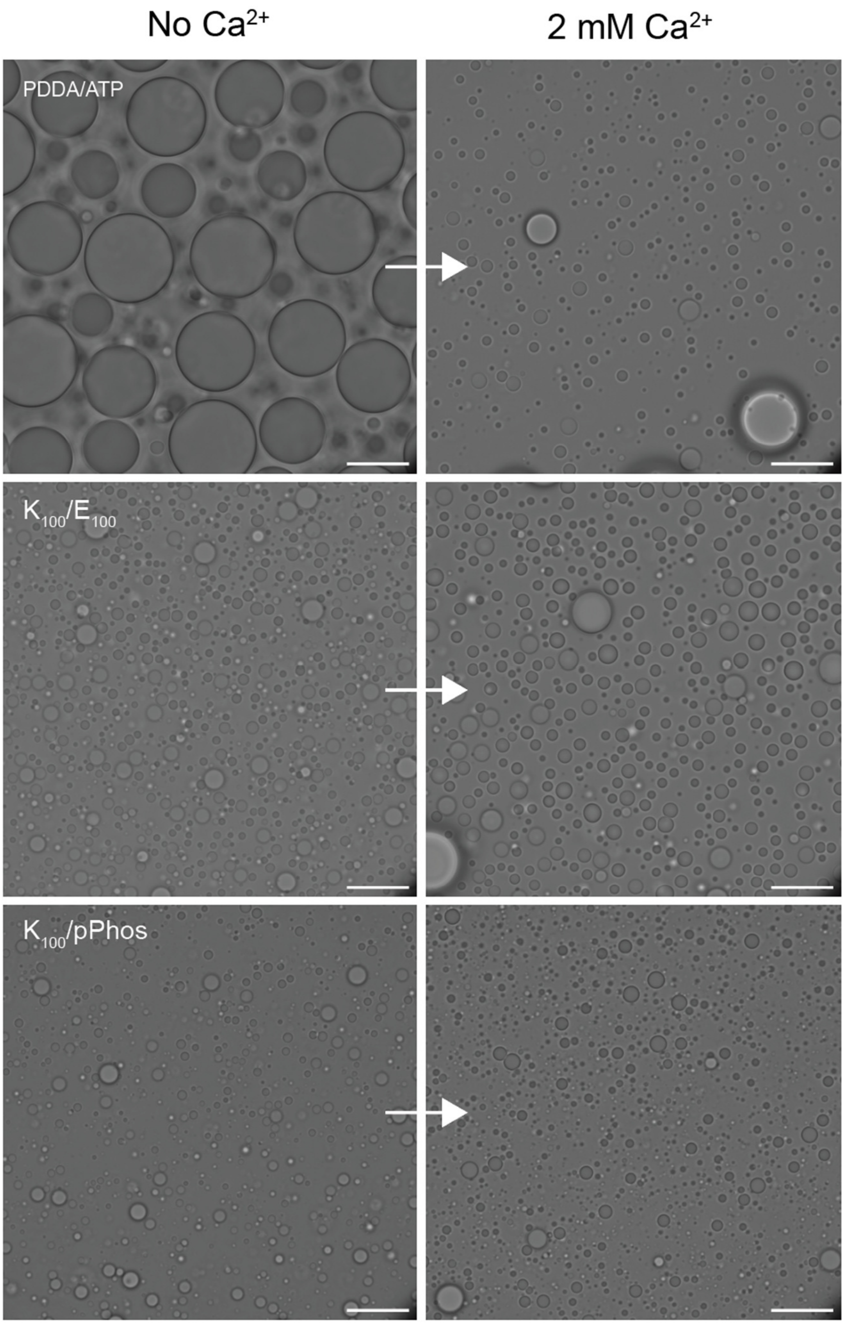

Figure S1: Effect of divalent ions on coacervates. Images show representative coacervate solutions before and after adding 2 mM $CaCl_2$ at 22 °C. Ca^{2+} dissolved PDDA/ATP coacervates, breaking them into smaller droplets. However, for other coacervate systems used in the study, 2 mM $CaCl_2$ did not induce visible changes in coacervate morphology. Scale bar = 5 µm.

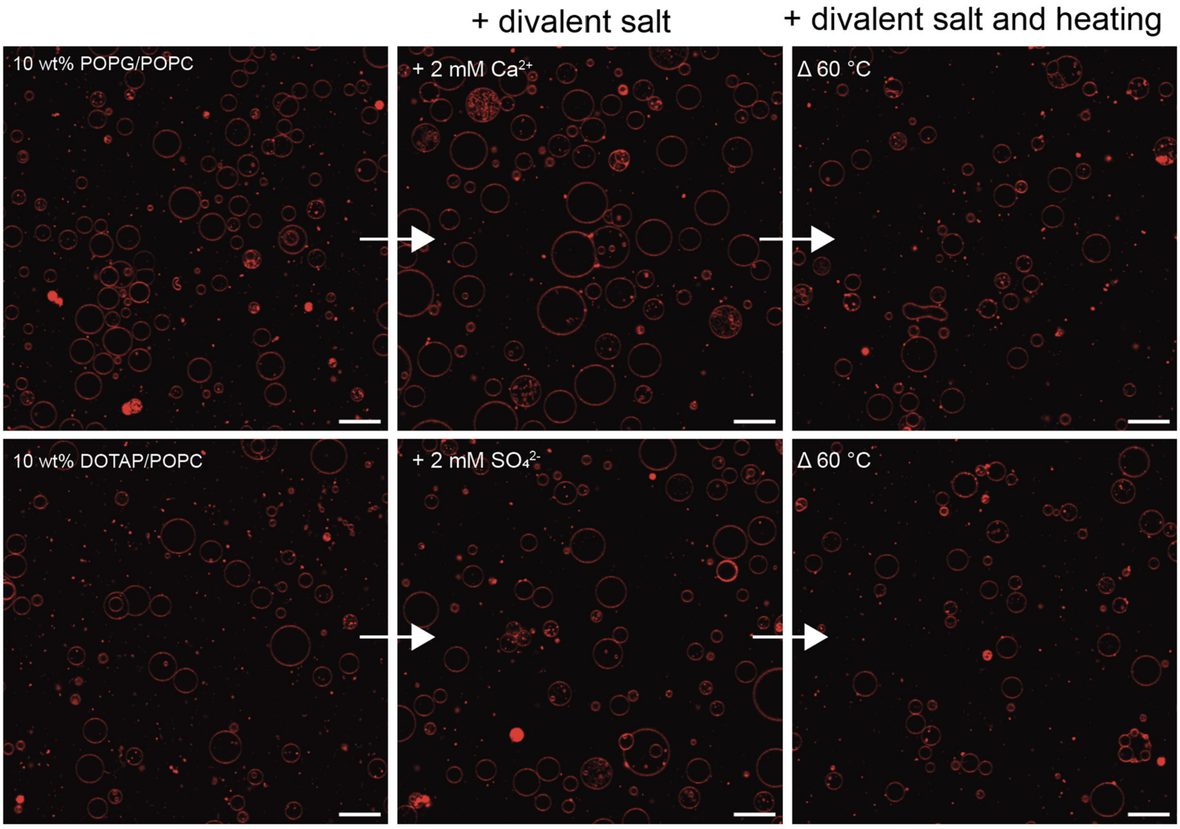

Figure S2: Effect of heating in presence of divalent ions on GUVs. Images show representative GUVs before and after heating till 60 °C in presence of divalent ions. GUVs stabilize themselves after cooling down from a short heat shock. We do not observe large morphological variations under between heated and unheated GUVs, indicating that the destabilization effect of high temperature exploited in MC formation is transient. Scale bar = 20 µm.

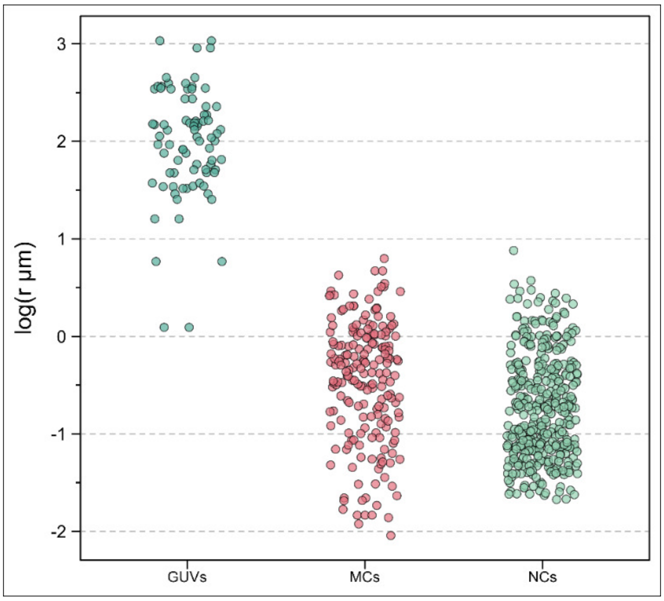

Figure S3: Size comparison for 20 wt% POPG/POPC GUVs, K_{100}/pPhos/K_{100}-FAM coacervates, and MCs formed with them. The size of the MCs is more comparable to that of the constituting coacervates rather than the GUVs, indicating that the final size of MCs depends on the size of the coacervates rather than the GUVs.

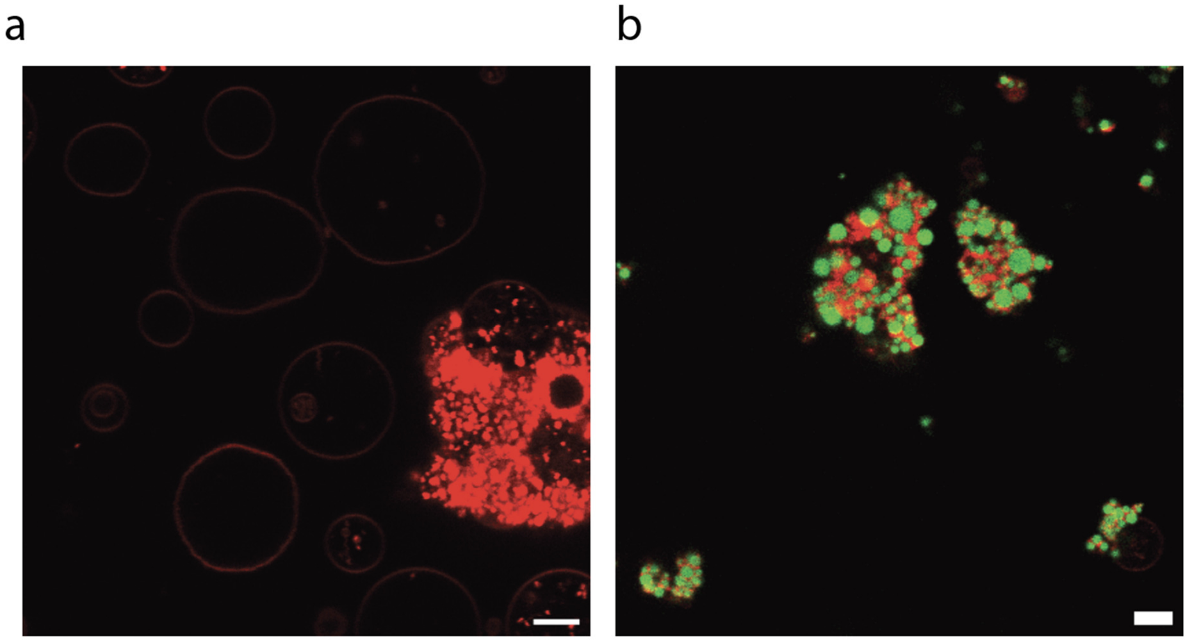

Figure S4: Lipid aggregates affect the quality of the final MCs. a) 20 wt%POPG/POPC GUV suspensions containing large lipid aggregates. b) K_{100}-pPhos MCs formed with the GUV suspension shown in a) form aggregates without a clear membrane around them. Scale bar = 5 µm.

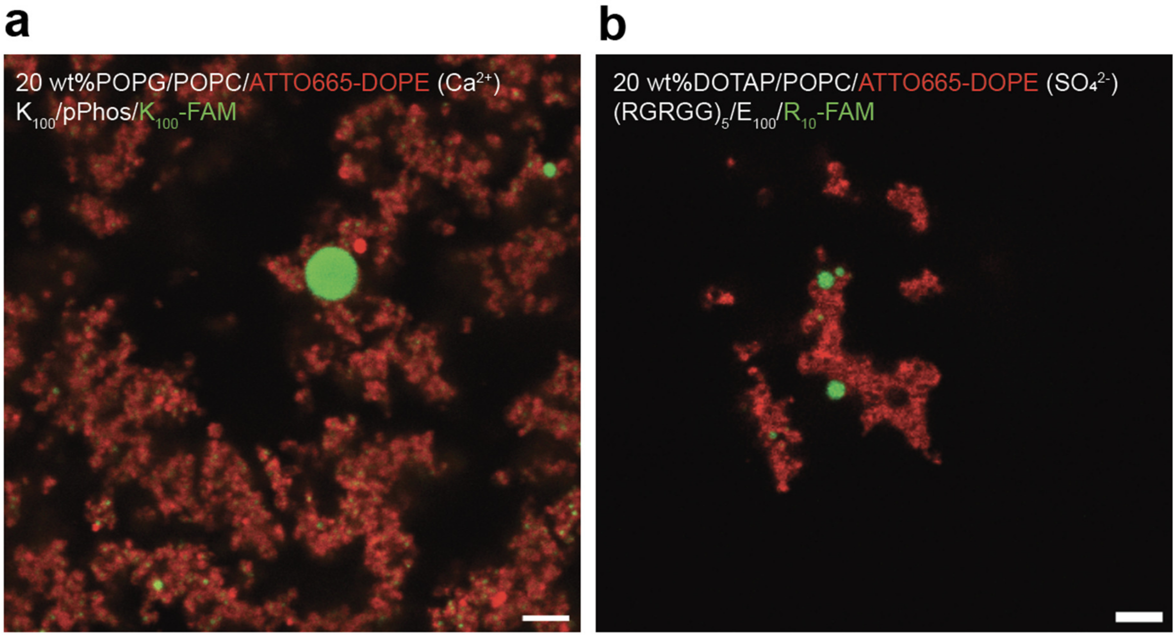

Figure S5: LUVs do not form membranized coacervates upon heating at 60 °C in presence of divalent ions. Mixing heat-destabilized LUVs with coacervates resulted in LUVs aggregating around coacervate material instead of forming membranized coacervates. a) Aggregates of LUVs constituting 20 wt%POPG/POPC and K_{100}/pPhos coacervates labelled with K_{100}-FAM. b) Aggregates of LUVs constituting 20 wt%DOTAP/POPC and $(RGRGG)_5$/E_{100} coacervates labelled with R_{10}-FAM. Both LUVs were labelled with ATTO665-DOPE. Scale bar = 5 µm.

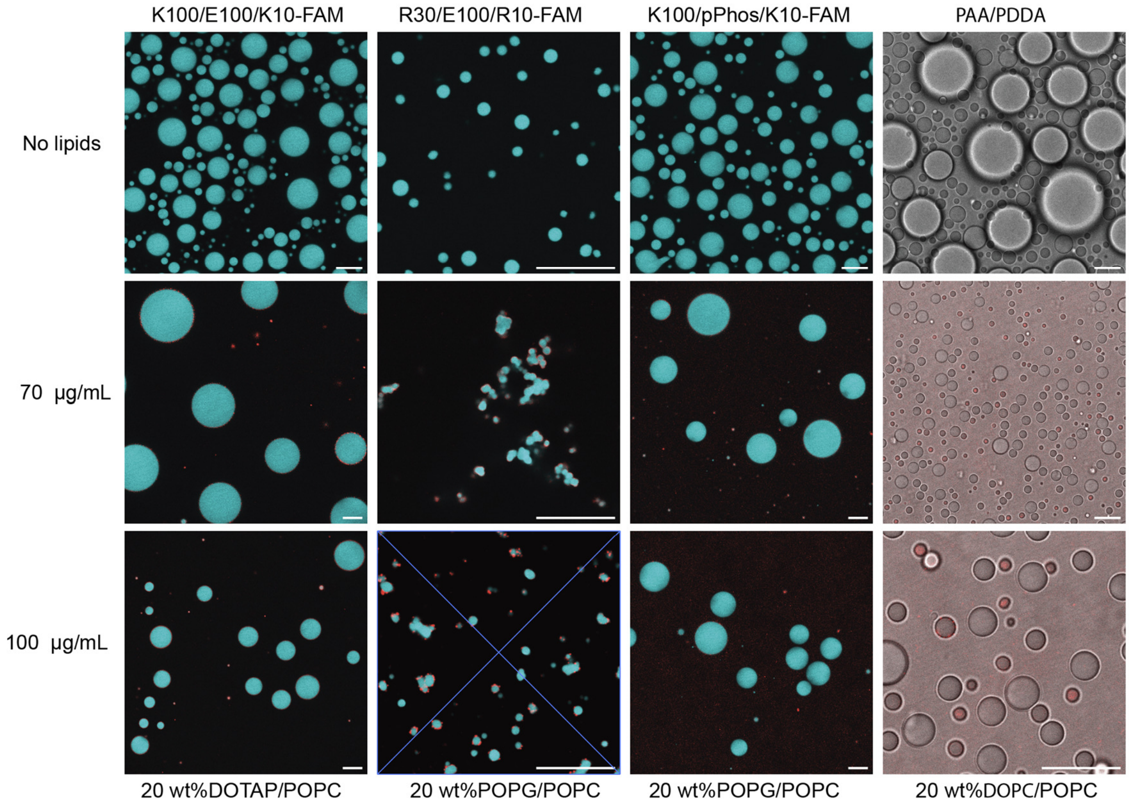

Figure S6: Adsorption of free lipids on coacervates. Adding lipids dissolved in ethanol to coacervate solution does not result in robust adsorption of the lipids on the coacervate surface. The lipids adsorption is visualized using ATTO633-DOPE in all cases. We tested four lipid-coacervate combinations that we also use to make membranized coacervates using GUVs (Table S1). The final ethanol content in the solution did not exceed 5% v/v. Even low amounts of ethanol affected the coacervates, either causing them to aggregate as in case of R30/E100 coacervates, dissolve as in case of PAA/PDDA coacervates, or coalesce rapidly as in case of K100/E100 and K100/pPhos coacervates. For PAA/PDDA coacervates, we also observed lipid partitioning inside the coacervates. Moreover, the composition of the lipids adsorbed on the coacervate surface cannot be accurately determined because we only visualize the labelled lipid. Adsorption of free lipids on the coacervate surface is also prone to high variability in the interfacial lipid composition across different experiments. Scale bar = 20 µm.

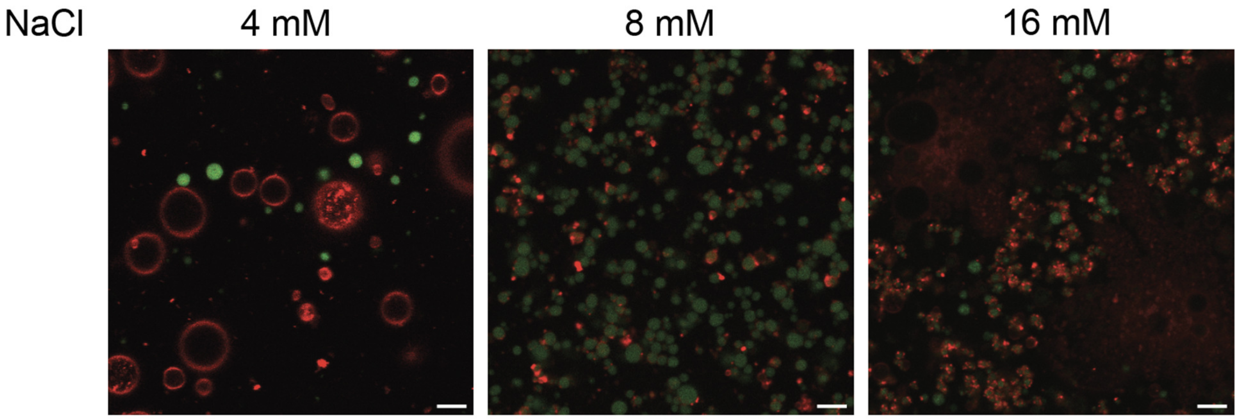

Figure S7: GUV membranes do not wrap around the coacervates in the presence of monovalent ions. The images show 20 wt%POPG/POPC GUVs and K_{100}/pPhos/K_{100}-FAM coacervates. There was no interaction between the coacervates and the GUVs after heating the GUVs at 60 °C in presence of 4 mM, 8 mM, or 16 mM NaCl. In presence of 16 mM NaCl, many GUVs burst, and the lipids spread on the microscopy slide instead of wrapping around the coacervates.

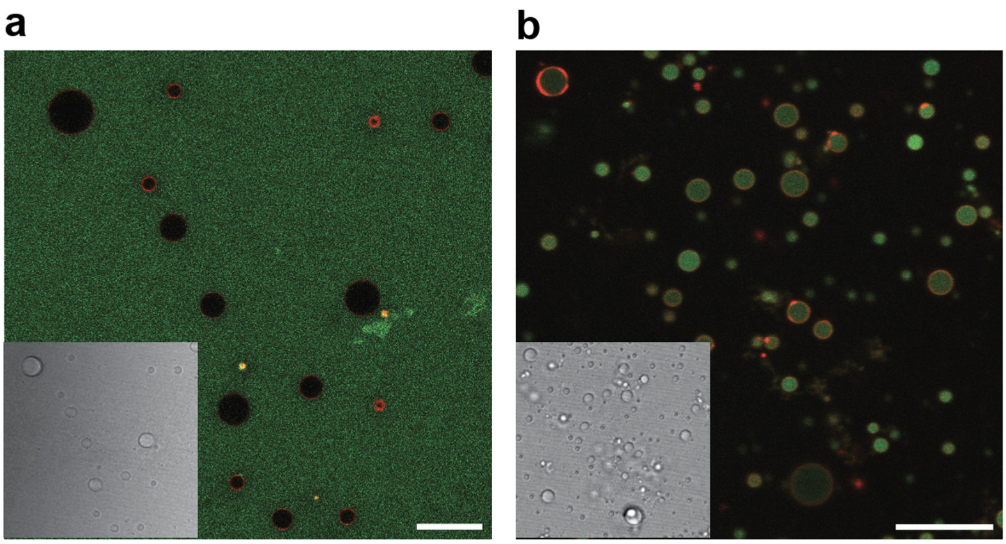

Figure S8: Melittin can permeabilize MC membranes. a) Membranized coacervates 20 wt% POPG/POPC-K_{100}/pPhos are impermeable to 5-TAMRA (10 µM). b) 5-TAMRA partitions inside the MCs after adding melittin (10 µg/mL), which can insert itself into MC membranes, form pores and permeabilize the membrane.

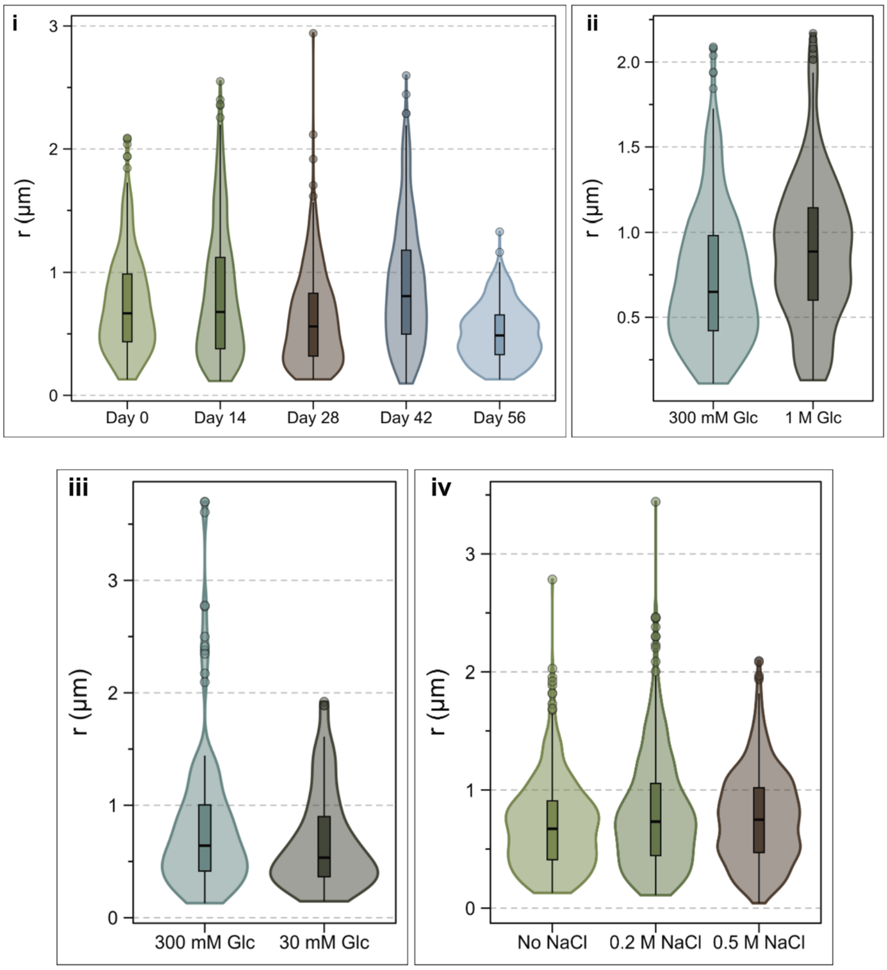

Figure S9: MC size distribution under different conditions. Size distribution of 20 wt% POPG/POPC-K_{100}/pPhos MCs after freeze-thaw cycles over 56 days (i), under hyper (ii) and hypoosmotic stress (iii), and in 200 mM and 500 mM NaCl solutions (iv).

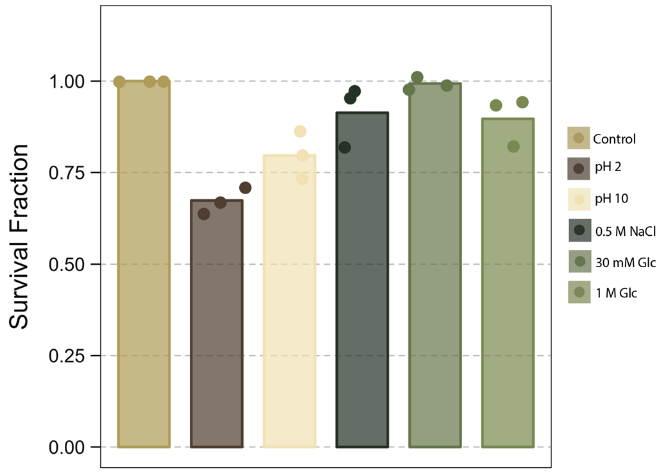

Figure S10: TAP/PC-K100/E100 MCs are resilient against fluctuations in the environment. We tested the resilience of TAP/PC-K100/E100 MCs at pH 2, pH 10, 0.5 M NaCl, 30 mM glucose, and 1 M glucose. The resilience of this MC system is different from that of the PG/PC-K100/pPhos MCs. This variable resilience and stability under stress conditions allows us to choose an MC system based on the application. Control = freshly prepared MCs without salt in 300 mM glucose solution at pH 7.4.

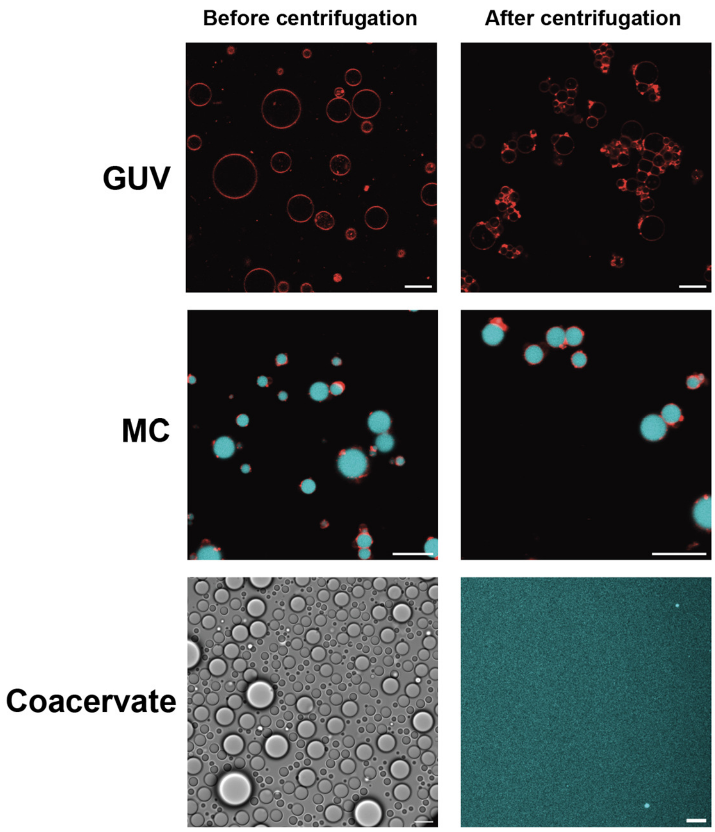

Figure S11: MCs resist coalescence. To check whether MCs coalesce as easily as coacervates do, we centrifuged 20 wt%POPG/POPC-K100/pPhos MCs at 1000 x g for 5 minutes and compared their state before and after centrifugation. We also did the same for 20 wt%POPG/POPC and K100/pPhos coacervates. To ensure similar conditions in across all samples, we added 2 mM $CaCl_2$ in the GUV and coacervate samples (as the MC sample contained the divalent salt). The MC pellet obtained after centrifugation can be resuspended by gentle mixing, much like the GUVs. The presence of divalent ions results in strong clustering of GUVs, likely due to screening of the negative charges between the vesicles by Ca^{2+}. Coacervates coalesce and cannot be resuspended into droplets just by gentle mixing. Scale bar = 10 µm.

Table S2: Flipper-TR™ lifetimes (ns) in 20 wt% PG/PC GUV and 20 wt%PG/PC-K100/pPhos MC membranes under different osmotic conditions. Data is presented as mean ± s.d.

	**10 wt% chol**			**20 wt% chol**		
	200 mM glucose	300 mM glucose	500 mM glucose	200 mM glucose	300 mM glucose	500 mM glucose
τ GUVs (ns)	3.16 ± 0.26	4.14 ± 0.12	5.9 ± 0.24	3.13 ± 0.42	4.74 ± 0.29	6.28 ± 0.5
τ MCs (ns)	4.97 ± 0.62	4.16 ± 0.25	5.36 ± 0.4	4.79 ± 0.71	5.1 ± 0.36	5.35 ± 0.54

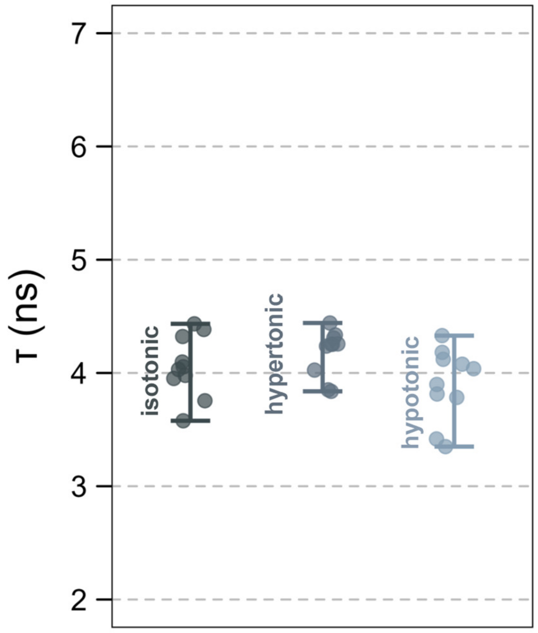

Figure S12: Flipper-TR™ lifetimes (ns) in 20 wt% DOTAP/POPC-K100/E100 MC membranes under different osmotic conditions. The mean ± s.d. lifetimes (ns) for the MC system isotonic (300 mM glucose), hypertonic (500 mM glucose), and hypotonic (200 mM glucose) conditions were 4.05 ± 0.26, 3.9 ± 0.3, 4.18 ± 0.2 ns, respectively. As for the 20 wt% POPG/POPC-K100/pPhos MCs (Fig. 6), we do not see a discernable difference in lipid packing under different osmotic conditions.

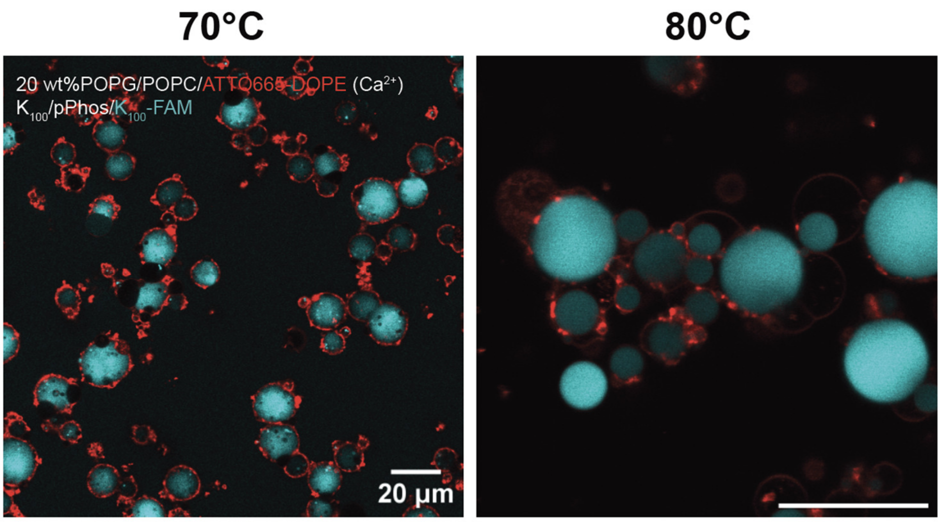

Figure S13: Destabilizing GUVs at higher temperatures. Heating 20 wt% POPG/POPC GUVs at 70 °C and 80 °C with 2 mM $CaCl_2$ does not result in ideally membranized coacervates. We hypothesize that at temperatures above 60 °C, the GUVs destabilize to a greater extent and much faster than they do at 60 °C. When coacervates are added to these GUVs, the membrane fragments, now more like aggregates, tend to deposit over the coacervate surface rather than wrap around them, resulting in thick lipid layer deposition on coacervates. Scale bar = 20 µm.

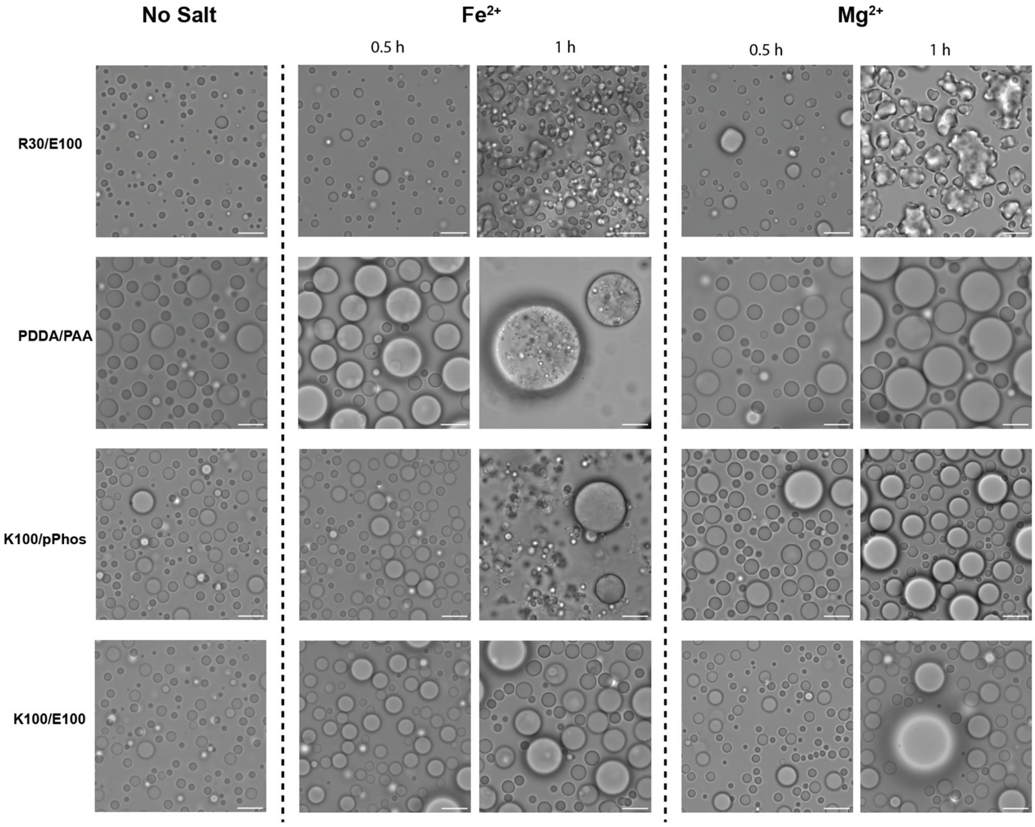

Figure S14: Effect of other divalent ions on coacervates. Fe^{2+} and Mg^{2+} (2 mM) have a different effect on coacervates than Ca^{2+} and SO_4^{2-}. Mg^{2+} accelerates aging of R30/E100 coacervates, while Fe^{2+} oxidizes to Fe^{3+} in solution and causes aggregation of coacervates over time. Scale bar = 10 µm.

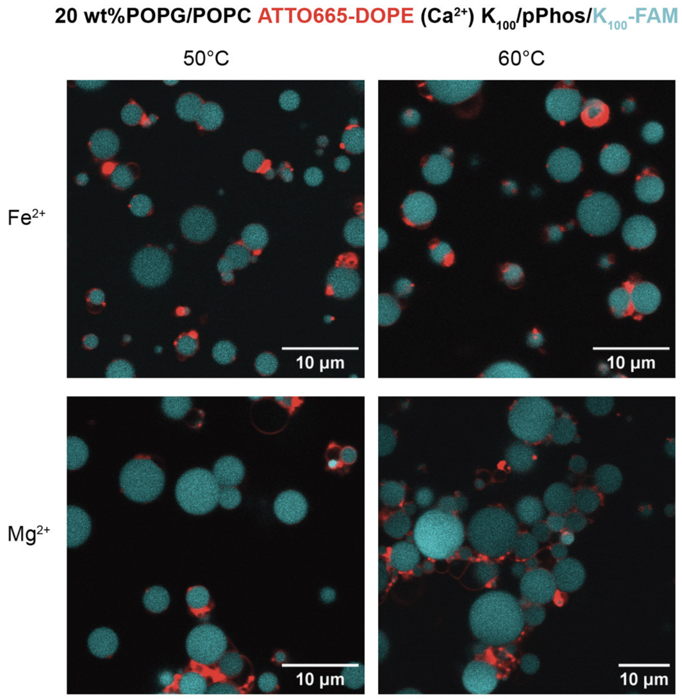

Figure S15: Making membranized coacervates using Mg^{2+} and Fe^{2+}. Heating 20 wt% POPG/POPC GUVs at 50 °C and 60 °C in presence of 2 mM of either Fe^{2+} or Mg^{2+} and adding K100/pPhos coacervates to the destabilized GUVs results in formation of partially membranized coacervates. The procedure to make membranized coacervates needs to be optimized for divalent ions other than Ca^{2+} and SO_4^{2-}.
